# Redheaded women are more sexually active than other women, but it is probably due to their suitors

**DOI:** 10.3389/fpsyg.2022.1000753

**Published:** 2022-11-29

**Authors:** Kateřina Sýkorová, Vojtěch Fiala, Jana Hlaváčová, Šárka Kaňková, Jaroslav Flegr

**Affiliations:** Department of Philosophy and History of Science, Faculty of Science, Charles University, Prague, Czechia

**Keywords:** redheadedness, sexual behavior, sexual desire, sexual activity, sexual submissiveness, stereotypes, mate selection

## Abstract

Women with red hair color, i.e., 1–9% of female Europeans, tend to be the subject of various stereotypes about their sexually liberated behavior. The aim of the present case–control study was to explore whether a connection between red hair color and sexual behavior really exists using data from 110 women (34% redheaded) and 93 men (22% redheaded). Redheadedness in women, correlated with various traits related to sexual life, namely with higher sexual desire as measured by Revised Sociosexual Orientation Inventory, with higher sexual activity and more sexual partners of the preferred gender over the past year, earlier initiation of sexual life, and higher sexual submissiveness. Structural equation modelling, however, showed that sexual desire of redheaded women mediated neither their higher sexual activity nor their higher number of sexual partners. These results indirectly indicate that the apparently more liberated sexual behavior in redheaded women could be the consequence of potential mates’ frequent attempts to have sex with them. Our results contradicted the three other tested models, specifically the models based on the assumption of different physiology, faster life history strategy, and altered self-perception of redheaded women induced by stereotypes about them. Naturally, the present study cannot say anything about the validity of other potential models that were not subjects of testing.

## Introduction

Human redheadedness is determined by the quantity, ratio, and distribution of the two main types of the pigment melanin: eumelanin and pheomelanin. In European populations, its expression is controlled mainly by the *MC1R* gene (Valverde et al., 1995; Lin and Fisher, 2007; Han et al., 2008). About 1–9% of Europeans have red hair, with the highest prevalence in Great Britain (Katsara and Nothnagel, 2019). Similar to other minorities, redheaded people are subjected to various stereotypes (Heckert and Best, 1997). Redheaded women are thought of as being more temperamental than other women (Weir and Fine-Davis, 1989; Heckert and Best, 1997; Swami and Barrett, 2011; Harvey, 2015; Ayres and Maier, 2021) and, unlike redheaded men, they are often stereotyped as sexy, passionate, sexually liberated, or promiscuous (Heckert and Best, 1997; O'Regan, 2014; Anderson, 2015; Harvey, 2015; Thornburg, 2020; Ayres and Maier, 2021). The existence of these stereotypes has been in the scientific literature documented only *via* indirect evidence, such as depictions of redheaded women in literature or the visual arts (Harvey, 2015; Ayres and Maier, 2021), eventually from interviews with individual respondents (Heckert and Best, 1997). In scientific literature, data regarding associations between red hair and traits related to sexual life are also nearly absent. There are only studies showing higher self-reported lifelong number of sexual partners in redheaded women (Frost et al., 2017), association between genetically predicted later age at first sexual intercourse and redheadedness in both sexes (Day et al., 2016), and no connection between self-reported sexual desire and redheadedness in either gender (Flegr and Sýkorová, 2019). All these papers, however, primarily focused on different topics. The nature and cause of a possible relation between red hair and sexual behavior in women therefore deserves a closer look.

Based on available scientific literature, we propose five principally distinct possible explanations of the potentially liberated sexual behavior in redheaded women. The hypothetical higher sexual activity of redheaded women could be either the result of their higher sexual desire or of a higher demand on the part of potential mates who prefer redheaded women or believe that redheaded women are more sexually permissive. If higher sexual desire of redheaded women were indeed responsible for their liberated sexual behavior, manifesting for instance in higher sexual activity or early initiation of sexual life, one would expect redheaded women to exhibit one of the following: (1) a difference in physiology, e.g., higher estrogen concentrations; (2) a faster life history strategy, possibly induced by worse health that would shorten life expectancy and likely reproductive period; (3) their altered self-perception with internalized belief in stereotypes about redheads. If the higher sexual activity of redheaded women were the result of higher demand for women with red hair, it could be either (4) a *direct* passive response of redheaded women to current courtship of potential mates, or (5) *indirect*, partly or fully active, response of redheaded women to past courtship of potential mates.

The first model supposes that redheaded women have on average higher sexual desire and sexual activity than non-redheaded women due to some molecular mechanism that affects both the expression of red hair in women and their sexual behavior. The most probable candidate is prenatal estrogen. It has been shown that women with a high 2D:4D digit ratio, which indicates exposure to a higher level of prenatal estrogen (Manning et al., 1998), report higher sex drive, higher sociosexual desire, and easier attainment of sexual excitement (Manning and Fink, 2008; Varella et al., 2014). Because red hair color generally seems to occur more frequently in women than in men (Shekar et al., 2008; Frost et al., 2017; Flegr and Sýkorová, 2019), Frost et al. (2017) suggested that prenatal estrogen, which is specific to female development, contributes to the expression of red hair during prenatal development and might be responsible for the sex difference in the frequency of redheadedness. Conversely, a study by Voracek et al. (2007) found that prenatal estrogen, as indirectly indicated by 2D:4D digit ratio, was not associated with lighter hair colors. Still, that study did not examine an association between estimated prenatal estrogen and red hair color specifically, because red hair color was treated merely as one of the dark colors of hair. The sex difference in expression of red hair phenotype is, however, supported by a twin study which showed that in four of five twin pairs discordant in red hair color, the females were redheaded and males non-redheaded (Box et al., 1997).

The second model suggests that higher sexual desire of redheaded women could be due to their switch from a slow to a faster life strategy, which could occur in response to poorer health. Redheaded people, especially women, tend to have worse health (Frost et al., 2017; Flegr and Sýkorová, 2019) and are more likely to suffer from certain diseases including cancer, endometriosis, and Parkinson’s disease (Bliss et al., 1995; Woodworth et al., 1995; Missmer et al., 2006; Gao et al., 2009; Scherer and Kumar, 2010; Tell-Marti et al., 2015; Chen et al., 2017). It has been described how in reaction to impaired health, which reduces adult life expectancy and therefore also the length of the reproductive period, individuals tend to shift to a faster life history strategy, which manifests itself as earlier reproduction or having a higher number of children (Waynforth, 2012; Chua et al., 2017). A recent study has moreover shown that poor health is associated not only with an earlier start of reproduction but also with an earlier initiation of sexual life in women and higher sexual desire in both women and men (Sýkorová and Flegr, 2021). At the same time, redheaded people have more children (Frost et al., 2017), which would suggest an explanation based on a faster life strategy. It is possible that the faster life strategy of redheaded people could be partly indicated even at a prenatal stage by some physiological parameters which signalize a higher likelihood of future worse health. It has been shown, after all, that the link between red hair and worse health has a genetic component (Han et al., 2006). Rh-negativity, another genetic factor predisposing to worse health, has also been suggested as a factor that can lead to adoption of a faster life strategy (Sýkorová and Flegr, 2021).

The third model proposes that redheaded women have higher sexual desire and are sexually more permissive because the stereotypes about their sexual behavior have altered their self-perception. In this process, an initially incorrect attribution assigned by others can be internalized by the target person who then changes their self-perception to conform to the initially erroneous belief (Snyder and Swann, 1978; Darley and Fazio, 1980; McNulty and Swann, 1994; Scherr et al., 2011). Based on such process of change in self-perception, redheaded women themselves could be internally convinced of the validity of the stereotypical social labelling, which would then lead to their actual higher sexual desire.

The fourth model puts potential mates, usually men, in the active role in explaining the association between women’s redheadedness and their higher sexual activity. If men preferred redheaded women or, based on prevailing stereotypes, anticipated greater likelihood of success in their attempts to have sex with women with red hair, they would do so relatively more frequently. It would automatically lead to a higher sexual activity and a higher number of sexual partners of redheaded women even if their sexual desire was average or even lower than average.

The existence of male preference for redheaded women and an evolutionary explanation for it have been suggested already by Frost (2006). According to his hypothesis, during the last ice ages in northern Europe, a shortage of males – caused by a higher male mortality rate in such a harsh environment – forced women to compete for mates. Under a higher pressure of sexual selection, the newly emerged light eye and hair colors ensured their female bearers higher levels of attention from potential mates and thus an advantage on the mating market. As a result, this rare-color advantage led to diversification of hair and eye coloring, including red hair phenotype. Frost et al. (2017) also assumed that the current frequency of hair colors in the population, where the prevalence of red hair is low, had stabilized and there is now an equilibrium between the rare-color advantage and the impaired health of people with red hair (Flegr and Sýkorová, 2019). Male preference for red, the rarest hair color, was not supported in several studies (Lawson, 1971; Clayson and Maughan, 1976; Feinman and Gill, 1978; Clayson and Maughan, 1986; Clayson and Klassen, 1989; Swami and Barrett, 2011; Guéguen, 2012). It was, however, partly supported by Wortham et al. (2018) who showed that red hair was preferred over other hair colors more frequently than expected based on the prevalence of redheads in the studied population (men preferred red hair 6% of the time, while only 3% of the female population were redheads).

The fifth model explains the higher sexual activity in redheaded women using the notion of social feedback (Kleisner et al., 2010). In particular, it proposes that if potential mates often tried to have sex with redheaded women on the first date or early on in the courtship, these women could start to view it as a social norm and consequently respond more positively to such behavior or even initiate sexual activity themselves. Because redheaded women would perceive such behavior as a social norm, they might be more sexually active even if they did not like it and were unfamiliar with stereotypes about more permissive redheaded women.

The main purpose of this case–control study was to explore whether there is an actual association between red hair color and traits related to sexual life and, if so, to seek a possible explanation. Specifically, we tested five models (see above) which could explain the existence of this association. During the study, we collected data about hair color, sexual behavior, and sexual preferences from 110 women and 93 men with various intensity of redheadedness. We analyzed the association of redheadedness with sexual desire, sexual activity, the number of sexual partners, age at first sexual intercourse, sexual orientation, BDSM (bondage-discipline, dominance-submission, and sadism-masochism) preferences, and sexual dominance. In an attempt to test several hypotheses that could explain the mechanism of the observed associations, we searched for a mediating role of sexual desire and physical disease.

## Materials and methods

The study consisted of a laboratory investigation which took place at the Faculty of Science of Charles University in Prague on September 17, 2018 to October 3, 2018 and subsequent online questionnaire survey performed with the same set of participants within the following few weeks. Information about the experimental design was already described in a study by Flegr et al. (2020b). The project was approved by the institutional review board of the Faculty of Science, Charles University (No. 2018/30).

### Participants

The recruitment of participants was conducted mostly *via* Facebook. The study was promoted as a “study of health and personality of redheads” on the timeline of the Facebook page Labbunnies, an approximately 18,000-strong group of Czech and Slovak nationals willing to participate in evolutionary psychology experiments. Anyone could share the link to invitation to the study. Further recruitment of redheaded participants was carried out by invitations on other Facebook pages, selective invitation of registered members of Labbunnies who reported having red hair in our earlier questionnaires (scored 4–6 on a 6-point scale of redheadedness), and by handing out flyers in the streets of Prague to people who looked like natural redheads. We invited only people who did not have graying hair and had not dyed or bleached their hair for at least 6 months. The ratio of redheaded participants to controls was intended to be 1:2. In the end, we obtained data from 110 women and 93 men who participated in the study. All provided informed consent. Due to the sensitivity of questions posed to them, participants took part in the study under assigned anonymized codes and were informed about the complete anonymization of the data. Participants received no remuneration, only a commemorative badge and a haircare gift set (costing 53 CZK, i.e., app. 2.3 USD). The data collection procedure and experimental design, as well as the method of computing aggregated variables, were preregistered prior to the beginning of the study on the website of the Open Science Framework.^1^

### Variables

In the project, redheadedness was determined using several methods: participants’ self-report in the questionnaire, experimenters’ observer report during the laboratory investigation, and two methods of measuring hair pigmentation with a spectrophotometer. A previously published study on the effects of redheadedness on vitamin D concentration, which was performed on the same data, showed that all the above-mentioned indicators of redheadedness correlated very strongly with each other and provided almost identical results (Flegr et al., 2020b). In the present study, we have therefore decided to employ only self-reported redheadedness because this will provide better comparability with various past (Frost et al., 2017; Flegr and Sýkorová, 2019) and future studies about the effects of redheadedness. Self-reported redheadedness was measured as a response to assessing current natural redheadedness on a six-point scale anchored with “absolutely non-red” (code 1) and “bright red” (code 6). Correlations between other indicators of redheadedness and sexual behaviors are presented in Supplementary Table S1, and again show that all methods of determining redheadedness provide highly similar results. It suggests that the simplest and cheapest method, namely participants’ self-rating using a short ordinal scale, should be preferred in future studies because it facilitates acquisition of data from large population samples. Measurement scales and methods of calculating other indicators of participants’ redheadedness, which are presented in the Supplementary Material, are described in Flegr et al. (2020b). Solely for the purposes of descriptive statistics, we have also calculated a binary variable from self-reported redheadedness, where “non-redheaded” corresponds to responses 1, 2, and 3 and “redheaded” corresponds to responses 4, 5, and 6.

All output variables were obtained from the questionnaire data. Sexual desire was computed as the arithmetical mean of Z-scores of three items forming the Desire facet in the Revised Sociosexual Orientation Inventory (Penke and Asendorpf, 2008), namely the frequency of having fantasies about having sex with someone the respondent is not in a committed romantic relationship with, the frequency of experiencing sexual arousal when the respondent is in contact with someone who they are not in a committed romantic relationship with, and the frequency of having spontaneous fantasies about having sex with someone the respondent had just met (each item has been anchored with “never” – code 1, and “at least once a day” – code 9). In the dataset of Penke and Asendorpf (2008), item loadings on the “desire” factor were > 0.74 and reliability of the items was α > 0.85. Sexual activity was estimated as the average number of sexual intercourse per month in the past year (eight categories: “0” – code 1, “1” – code 2, “2–3” – code 3, “4–6” – code 4, “7–10” – code 5, “11–20” – code 6, “21–30” – code 7, “over 30” – code 8). Non-heterosexuality was calculated from two variables that inquired about the intensity of being attracted to people of the same and the opposite sex (anchored with “absolutely not” – code 0, and “absolutely yes” – code 100; we intended to exclude any subjects who would respond with “0” on both scales but no such subject took part in the study). Non-heterosexuality was treated as a binary variable with 1 corresponding to being sexually attracted to people of the same sex with the same or higher intensity than to people of the opposite sex. In heterosexuals, we assessed the number of sexual partners of the preferred sex as the number of sexual partners of the opposite sex in the past year (nine categories: “0” – code 1, “1” – code 2, “2” – code 3, “3” – code 4, “4” – code 5, “5–6” – code 6, “7–9” – code 7, “10–19” – code 8, “20 or more” – code 9). In non-heterosexuals, the number of sexual partners of the preferred sex was expressed as the higher code of two variables assessing the number of female and male sexual partners in the past year (on the same scale as above). Age at first sexual intercourse was determined as age at such activity (restricted from below with “12 and less” – code 12 and restricted from above with “40 and more” – code 40; “I have not had sex yet.” corresponded to code 0). BDSM index was calculated as the arithmetical mean of Z-scores of responses to questions concerning sexual preferences, namely the arousal by violence, own pain, placing oneself in danger, own powerlessness, own humiliation, other’s pain, being in danger, powerlessness, and humiliation (each item was anchored with “absolutely not” – code 0, and “absolutely yes” – code 100). Index of sexual dominance was computed as the arithmetical mean of Z-scores of 4 differences between arousal by other’s and own pain, being in danger, powerlessness, and humiliation.

We also examined a number of potentially confounding variables including sex, age, size of place of residence (six categories: “<1,000 inhabitants”, “1,000–5,000”, “5,000–50,000”, “50,000–100,000”, “100,000–500,000”, “Czech or Slovak capital”) and current sexual partnership status (having a stable sexual partner, binary variable). Physical disease was calculated as the arithmetical mean of Z-scores of responses to the number of antibiotic treatments a respondent used in the past year, the number of different kinds of drugs prescribed by a medical doctor which a respondent uses daily, the number of different kinds of non-prescription drugs or food supplements a respondent uses on a daily basis, the number of visits to a general practitioner in the past year, the number of different medical specialists a respondent visited in the past year (each item had 1–9 scale), and the intensity of physical health problems (0–100 scale). Similarly, mental disease was computed as the arithmetical mean of Z-scores of responses to the intensity of suffering from anxieties, depressions, manias, obsessions, phobias, visual hallucinations, auditory hallucinations, burnout, and headaches, and the intensity of mental health problems (each item had 0–100 scale). Physical and mental disease were calculated from variables used for the computation of indices of physical and mental health problems in several previously published studies (Flegr and Horáček, 2020; Flegr et al., 2020a). Variables pertaining to current health status were selected by a general practitioner (Flegr et al., 2015).

### Statistical analysis

Before statistical analyses, we filtered out records of three subjects who had gray hair or did not provide information about their age. When a respondent omitted over 20% of questions relevant for one index (i.e., sexual desire, BDSM index or index of sexual dominance), we did not compute the index for this subject and excluded their data from particular tests. In case missing data accounted for under 20% of variables relevant for a particular index, that index was calculated from the remaining variables. The percentage of excluded cases from the tests including sexual desire, BDSM index, and the index of sexual dominance were 1, 12, and 11%, respectively.

The age of women and men was compared using the Wilcoxon test. Associations of all focal variables with sex and age were examined by the non-parametric Kendall correlation test. Associations of all focal variables with potentially confounding variables (i.e., size of place of residence, current sexual partnership status, physical disease, mental disease) were analyzed by a partial Kendall correlation test with age as a covariate.

In theory, the effect of redheadedness on traits related to sexual life need not apply only to women. Therefore, we have initially fitted generalized linear models (GLM) with redheadedness, sex, age, and interaction between redheadedness and sex as predictors. Redheadedness was set as an ordered categorical predictor, while sex was a binary variable and age was on a pseudo-continuous scale. Each dependent variable was ascribed to a family based on a visual inspection of density plots and histograms. We have also considered the distribution that would be most likely based on the expected data-generating process. For example, in case of the number of sexual partners of the preferred sex, we expected this variable to demonstrate a Poisson distribution. In the case of non-heterosexuality, we expected the variable to be binomially distributed. To include the effect of subjects who reported not having had their first sexual intercourse yet, we conducted a survival analysis, namely the Cox regression (where “still alive” equals “still a virgin”). Prior to the Cox regression, independent variables were standardized by computing Z-scores and redheadedness was set as ordinal. The Cox regression model also included redheadedness, sex, interaction redheadedness–sex, and age as predictors.

Because the tested hypothesis about the effect of redheadedness on traits related to sexual life concerned women, we have subsequently analyzed women and men separately. We tested associations between redheadedness and traits related to sexual life using a partial Kendall correlation test with age as a covariate. In the next step, we used the same test with age and potentially confounding variables that had a significant effect on the output variables as covariates.

To investigate the role of potentially mediating variables in the association between redheadedness and sexual behavior, we performed structural equation modelling, in particular path analyses. Prior to path analyses, multivariate normality of data was tested by Mardia’s test. Since the data was non-normally distributed, and redheadedness, sexual activity, and the number of sexual partners of the preferred sex were set as ordinal, parameters were estimated using the diagonally weighted least square (DWLS) estimator. When comparing nested models, we considered changes in fit indices, such as the comparative fit index (CFI) and the root mean square error of approximation (RMSEA). To establish invariance between models, the following criteria had to be matched: ΔCFI < −0.005 and ΔRMSEA <0.010 (Chen, 2007).

To assess the strength of the observed effects, we used the widely accepted borders by Cohen (1977). After transformation between τ and d, τ 0.062, 0.156, and 0.241 correspond to d 0.20 (small effect), 0.50 (medium effect), and 0.80 (large effect), respectively (Walker, 2003). For the main tests, sensitivity power analyses were performed where a bivariate normal model (two-tailed test) was used as an approximation of Kendall correlation test and power (1- β) was set to 0.80. To address the issue of multiple testing, we applied the Benjamini–Hochberg procedure with false discovery rate set at 0.1 to the set of partial Kendall correlation tests. Statistical analysis was performed with R v. 4.1.1 using packages “fitdistrplus” 1.1.8 (Delignette-Muller and Dutang, 2015) for initial inspection of distributions of the dependent variables, “Explorer” 1.0 (Flegr and Flegr, 2021), “corpcor” 1.6.9 (Schäfer and Strimmer, 2005; Opgen-Rhein and Strimmer, 2007), and “pcaPP” 1.9.73 (Croux et al., 2007, 2013) for analyses with the partial Kendall correlation test, “survival” 3.4.0 (Therneau, 2020) for computing Cox regression, “mvnormalTest” 1.0.0 (Zhou and Shao, 2014) for using Mardia’s test, and “lavaan” 0.6.12 (Rosseel, 2012), and “semPlot” 1.1.6 (Epskamp, 2015) for conducting the path analysis. Sensitivity power analyses were conducted using G*Power v. 3.1 (Faul et al., 2007). The dataset used in this article can be accessed on Figshare at https://doi.org/10.6084/m9.figshare.21200968.v1. R script containing the GLMs, Cox regression and path analyses is likewise published on the Figshare at https://doi.org/10.6084/m9.figshare.21201004.v1.

## Results

The final set consisted of 109 women (mean age 27.3, SD = 7.5) and 91 men (mean age 31.8, SD = 8.8). The age of women and men differed significantly (W = 3.458, *p* < 0.001). On a six-point scale of self-reported redheadedness, 37 (33.9%) women and 20 (22%) men responded 4 and higher. Descriptive statistics for all variables are presented in Supplementary Table S2.

We computed correlations of redheadedness and output variables with sex and potentially confounding variables (age, size of place of residence, current sexual partnership status, physical disease, and mental disease). Many focal variables significantly correlated with sex and age. For instance, women had more intensively red hair than men and redheadedness correlated negatively with age. Regarding other potential covariates, current sexual partnership status was significantly associated with four out of seven variables related to sexual behavior. Size of place of residence, physical disease, and mental disease did not exhibit many significant associations with predictors and output variables (see Supplementary Table S3).

Initially, we analyzed the effect of redheadedness on variables related to sexual life with GLM. In the first model for sexual desire, there was a significant linear effect of redheadedness and a significant effect of sex. Sexual desire was significantly higher in men (β = 0.529, SE = 0.148, *p* < 0.001) and associated positively with redheadedness (β = 0.506, SE = 0.223, *p* = 0.025). Interaction between redheadedness (linear trend) and sex was not significant in this model (β = −0.476, SE = 0.360, *p* = 0.187). In the second model for sexual activity, redheadedness also had a significant linear effect (β = 1.653, SE = 0.549, *p* = 0.003). There also was a significant interaction between the linear effect of redheadedness and sex (β = −1.872, SE = 0.879, *p* = 0.035), suggesting that the overall positive effect of redheadedness on sexual activity is driven mainly by the effect in women (Supplementary Figure S1). In the third model, the number of sexual partners of the preferred sex was significantly predicted by redheadedness (β = 0.570, SE = 0.150, *p* < 0.001, Poisson GLM fitted). The increase in redheadedness was associated with a linear increase of 1.77 in the number of sexual partners of the preferred sex. In this model, there was no significant interaction between redheadedness (linear trend) and sex (β = −0.250, SE = 0.246, *p* = 0.310). In the fourth model, which modelled non-heterosexuality, none of the predictors (including redheadedness, sex, age, and redheadedness–sex interaction) was significant (β = −10.511, SE = 2333.313, *p* = 0.996 for the linear effect of redheadedness and β = 13.172, SE = 2383.974, p = 0.996 for the redheadedness–sex interaction). In the fifth model, the index of sexual dominance was significantly predicted only by sex (β = 0.573, SE = 0.160, *p* < 0.001), suggesting that men are more dominant. In this model, we have also observed a non-significant linear trend of the association between redheadedness and the index of sexual dominance (β = 0.401, SE = 0.239, *p* = 0.096). The interaction between redheadedness (linear trend) and sex was not significant (β = 0.186, SE = 0.387, *p* = 0.632). The model for BDSM index revealed no significant effect of any predictor (β = 0.164, SE = 0.230, *p* = 0.477 for linear effect of redheadedness, β = −0.494, SE = 0.372, *p* = 0.185 for the redheadedness–sex interaction).

In our dataset, 10 (9.2%) women and five (5.5%) men reported they had not yet had their first sexual intercourse. We have thus performed a Cox regression to include the effect of those subjects. Redheadedness (as a linear trend) showed a significant effect on the likelihood of having first sexual intercourse (HR = 2.527, *p* = 0.002). There was also a significant positive quartic (^4) spline in the model (HR = 2.580, p = 0.003), suggesting that the association between age at first sexual intercourse and redheadedness was not linear and increased with a higher level of redheadedness. The interaction between redheadedness (linear trend) and sex was not significant in this model (HR = 0.494, SE = 0.472, *p* = 0.135).

The GLM found significant effects of redheadedness on certain variables related to sexual life and in one model also a significant effect of the redheadedness–sex interaction. Therefore, we have subsequently also analyzed women and men separately with partial Kendall correlation controlled for age. In women, redheadedness positively correlated with sexual desire (τ = 0.233, *p* < 0.001, *d* = 0.766), sexual activity (τ = 0.306, p < 0.001, *d* = 1.045), and the number of sexual partners of the preferred sex (τ = 0.286, p < 0.001, *d* = 0.965), and negatively with age at first sexual intercourse (τ = −0.145, *p* = 0.039, *d* = 0.465) and the index of sexual dominance (τ = −0.158, *p* = 0.023, *d* = 0.506). These results represent strong and medium effects (Cohen, 1977; Walker, 2003) (also see Materials and methods). Redheadedness in women did not show a significant correlation with non-heterosexuality (τ = −0.053, *p* = 0.434, *d* = 0.166) or with the BDSM index (τ = 0.102, *p* = 0.141, *d* = 0.323).

In men, redheadedness showed no significant correlation with any variable related to sexual life (sexual desire: τ = 0.066, *p* = 0.362, *d* = 0.208; sexual activity: τ = 0.088, *p* = 0.226, *d* = 0.278; the number of sexual partners of the preferred sex: τ = 0.102, *p* = 0.197, *d* = 0.324; age at first sexual intercourse: τ = 0.082, *p* = 0.273, *d* = 0.259; non-heterosexuality: τ = 0.063, *p* = 0.424, *d* = 0.199; BDSM index: τ = 0.110, *p* = 0.181, *d* = 0.349; index of sexual dominance: τ = 0.035, *p* = 0.676, *d* = 0.111). The results for both sexes remained qualitatively the same after correction by Benjamini-Hochberg procedure.

Current sexual partnership significantly correlated with sexual activity, the number of sexual partners of the preferred sex, non-heterosexuality, and BDSM index (Supplementary Table S3). We have therefore repeated the analysis with partial Kendall correlation controlled for age and current sexual partnership. All results for women retained significance (sexual desire: τ = 0.238, *p* < 0.001; sexual activity: τ = 0.334, *p* < 0.001; the number of sexual partners of the preferred sex: τ = 0.281, *p* < 0.001; age at first sexual intercourse: τ = −0.146, *p* = 0.039; index of sexual dominance: τ = −0.157, *p* = 0.025). For men, the results remained non-significant.

Sensitivity power analysis for the female set (with the lowest N of performed tests, i.e., N = 94) showed that the smallest detectable effect was Pearson’s r = 0.28, which corresponds to Kendall’s τ = 0.18. For the male set (with the lowest N of performed tests, i.e., N = 67), the smallest detectable effect was Pearson’s r = 0.33, which corresponds to Kendall’s τ = 0.21.

The higher sexual activity of redheaded women could either result from their own higher sexual desire or be the consequence of potential mates’ higher demand for redheaded women (who are either found by men to be more appealing or believed to be more sexually permissive). If the former is true, and higher sexual activity of redheaded women is the result of their own higher sexual desire, then it should be mediated by their higher sexual desire, while if the latter is true, sexual desire does not play a mediating role and the intensity of sexual activity in redheaded women is not associated with a higher intensity of sexual desire on their part. To discriminate between these two possibilities, we studied the effect of redheadedness on sexual activity and the number of sexual partners of the preferred sex using path analyses with sexual desire as a potentially mediating variable. In order to also test the hypothesis about higher sexual desire of redheaded women being a part of a faster life strategy (see Introduction), we included physical disease and age into the structured models.

**Figure 1:**
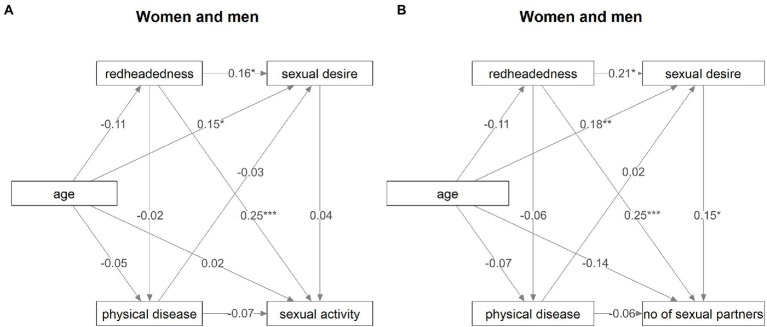
Direct and indirect effects of redheadedness on sexual activity and the number of sexual partners of the preferred sex. The figure visualizes the results of path analyses. Numbers at arrows show standardized parameter estimates. Associations with *p* values under 0.05 are marked by “*”, those with p values under 0.01 are marked by “**”, and those with p values under 0.001 are marked by “***”. **(A)** Relations between redheadedness and sexual activity in both women and men. **(B)** Relations between redheadedness and the number of sexual partners of the preferred sex in both women and men.

In women and men considered together, redheadedness had a direct positive effect on both sexual desire and sexual activity (Figure 1A). The direct effect of redheadedness on sexual activity (standardized parameter estimate = 0.25) was stronger than the indirect (sexual desire-mediated) effect on sexual activity (standardized parameter estimate = 0.16 * 0.04 = 0.006). The direct effect of redheadedness on sexual activity also was stronger than the direct effect of redheadedness on sexual desire (standardized parameter estimate = 0.16). Similarly, redheadedness had a direct positive effect on both sexual desire and the number of sexual partners of the preferred sex (Figure 1B). The direct effect of redheadedness on the number of sexual partners of the preferred sex (standardized parameter estimate = 0.25) was likewise much stronger than the significant indirect effect of redheadedness (standardized parameter estimate = 0.21 * 0.15 = 0.031). Alternative, more parsimonious models which did not include direct paths from redheadedness to either sexual activity or the number of sexual partners of the preferred sex showed a worse fit (model with sexual activity: ΔCFI = −0.70, ΔRMSEA = 0.24; model with the number of sexual partners of the preferred sex: ΔCFI = −0.34, ΔRMSEA = 0.24), in fact an inadequate fit to the data. Path analysis also showed no effect of redheadedness on physical disease, nor any effect of worse health on sexual desire, sexual activity, or the number of sexual partners of the preferred sex. Consequently, our findings do not support the hypothesis that higher sexual desire in redheaded people is driven by adoption of a faster life strategy.

**Figure 2:**
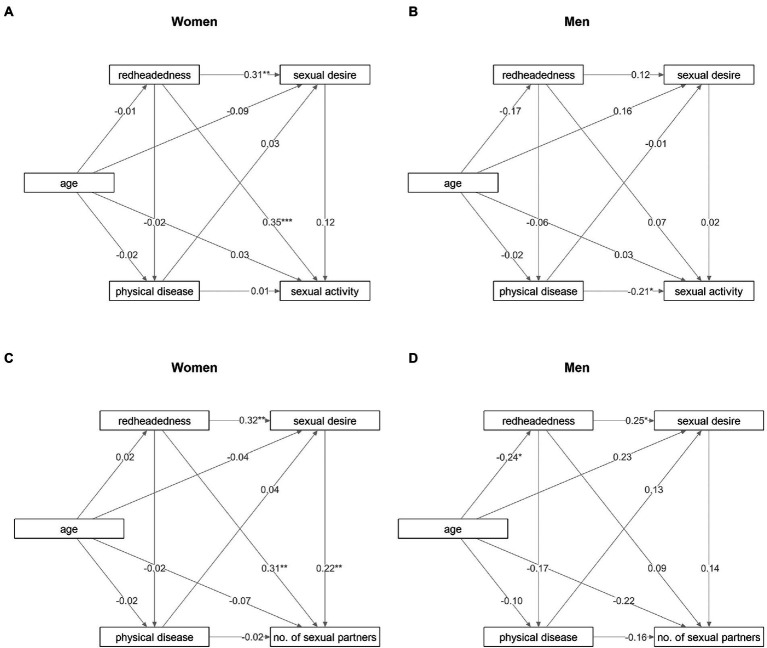
Direct and indirect effects of redheadedness on sexual activity and the number of sexual partners of the preferred sex in women and men. The figure visualizes the results of path analyses. Numbers at arrows show standardized parameter estimates. Associations with p values under 0.05 are marked by “*”, those with p values under 0.01 are marked by “**”, and those with p values under 0.001 are marked by “***”. **(A)** Relations between redheadedness and sexual activity in women. **(B)** Relations between redheadedness and sexual activity in men. **(C)** Relations between redheadedness and the number of sexual partners of the preferred sex in women. **(D)** Relations between redheadedness and the number of sexual partners of the preferred sex in men.

Because the GLM revealed that the effect of redheadedness on sexual activity depends on sex, we have repeated the analyses separately for each sex. The models for women showed the same pattern as the models for both sexes (Figures 2A,C). Redheadedness in women also had direct positive effects on sexual desire, sexual activity, and the number of sexual partners of the preferred sex. The direct effect of redheadedness on sexual activity (standardized parameter estimate = 0.35) was likewise stronger than the indirect (sexual desire-mediated) effect on sexual activity (standardized parameter estimate = 0.31 * 0.12 = 0.04). Similarly, the direct effect of redheadedness on the number of sexual partners of the preferred sex (standardized parameter estimate = 0.31) was stronger than the indirect effect on the number of sexual partners of the preferred sex (standardized parameter estimate = 0.32 * 0.22 = 0.07). These results indicate that neither the higher sexual activity nor the higher number of sexual partners of the preferred sex are driven by redheaded women’s higher sexual desire. In men, redheadedness had a direct positive effect on sexual desire in the model for the number of sexual partners of the preferred sex (Figure 2D), but this effect did not reach statistical significance in the model for sexual activity (Figure 2B; *p* = 0.28). Path analysis showed no effect of redheadedness on physical disease in either sex, and the data thus provided no support for the hypothetical explanation based on adoption of a faster life strategy. Worse health had a negative impact on sexual activity only in men.

## Discussion

The results of this study showed that the intensity of redheadedness is associated with shifts in sexual behavior, especially in women. Women with red hair color exhibited higher sexual desire and sexual activity, a higher number of sexual partners of the preferred sex, earlier initiation of sexual life, and lower sexual dominance (and therefore higher sexual submissiveness). However, one model for men also showed a positive effect of redheadedness on sexual desire.

Our results are in accordance with recent findings of Frost et al. (2017), who reported a higher lifelong number of sexual partners in redheaded women. Similarly, our results correspond to the results of two studies that showed no relationship between hair color (including red) and sexual orientation (Loehlin and McFadden, 2003; Ellis et al., 2008). Contrary to our results, a large internet study by Flegr and Sýkorová (2019) found no significant correlation between self-reported red hair color and sexual desire in either gender. In that study, however, sexual desire was measured as the intensity of being sexually attracted to people of the preferred sex (self-reported using a 1–100 scale), which might differ from sexual desire estimated more accurately by the SOI-R in the present study. Also inconsistent with our observations is a result by Day et al. (2016), who reported an association between genetically predicted red hair and later age at first sexual intercourse in both sexes. But that study involved subjects aged 40–69 years, while participants of the present study were at the peak of reproductive age, i.e., around 30. The above-mentioned difference in results could be thus due to the differences in participants’ age or due to the age-cohort effect. In future studies, these explanations should be tested on an older Czech population sample.

The higher sexual activity of redheaded women was not mediated by their higher sexual desire and the mediating effect of sexual desire in the association between redheadedness and number of sexual partners was weak. These observations thus do not support the first, second, and third model explaining sexual behavior in redheaded women (see Introduction). It would seem that the fact that women have red hair more frequently than men do and, according to our data, exhibit more liberated sexual behavior more often than men do might support the first model, which suggests the role of a molecule, e.g., prenatal estrogen, that would affect both women’s hair color and sexual behavior. This model also seems to find support in our results which show that redheaded women express higher sexual submissiveness, because the intensity of submissiveness is associated with a higher 2D:4D digit ratio that indicates exposure to prenatal estrogen (Manning and Fink, 2008; Butovskaya et al., 2015). But if prenatal estrogen were indeed responsible for higher sexual activity and the higher number of sexual partners in women with red hair, it would affect their sexual desire, which would then mediate, to a large extent, their liberated sexual behavior. The existence of such a substantial mediating role of sexual desire is, however, not supported by the results of the path analysis.

On the other hand, sexual desire is not the only one possible motive for active initiation of sexual activity (see limitations in Discussion below). It is well possible that higher sexual desire – in consequence of prenatal estrogen or another intrinsic factor, e.g., on the level of neurotransmitters or hormones – in combination with another motive for having sex specific to redheaded women could explain why redheaded women are more sexually active. This subject, however, requires further investigation.

Our results also do not support the second model, which proposes that sexual desire in redheaded women could be part of their faster life strategy induced by worse health. First of all, we found no correlation between redheadedness and physical disease in either sex and path analyses showed no mediation effect of worse health in associations between redheadedness and sexual desire. Secondly, redheaded women reported higher sexual activity and a higher number of sexual partners of the preferred sex, while a previous study has shown that the faster life strategy induced by impaired health results in a higher sexual desire but *lower* sexual activity and having fewer sexual partners (Sýkorová and Flegr, 2021). That study suggested that poor health adversely affects wellbeing and various physiological functions related to sexual life.

Faster life strategy is, however, adopted not only in response to worse health. It has been described many times that faster life strategy is predicted by exposure to harsh or unpredictable environments during childhood. This includes factors such as low socioeconomic status, dangerous environment during development, or unfavorable family circumstances and subsequent development of undesirable psychological traits (Bereczkei and Csanaky, 2001; Nettle, 2010; Griskevicius et al., 2011; Sheppard et al., 2016; Valentova et al., 2020; Figueredo et al., 2021). In view of the fact that redheaded people are often stigmatized or bullied for their hair color (Heckert and Best, 1997), it is possible that they might adopt a faster life strategy in reaction to such unfavorable conditions. This hypothesis, however, would need to be tested by further studies.

The data of the present study do, however, seem to indirectly support the fourth model, which suggests that the higher sexual activity and number of sexual partners in redheaded women are the result of their passive response to frequent mating efforts, usually made by men (who prefer redheaded women or believe stereotypes about permissive redheaded women). The stereotype about higher sexual permissiveness of redheaded women seems to be widespread among Czech and Slovak men (see Supplementary Table S4). In redheaded women, neither the higher sexual activity nor the higher number of sexual partners were primarily mediated by their higher sexual desire. In fact, the intensity of their sexual activity was relatively higher than the intensity of their sexual desire. This seems to suggest that it is not the redheaded women’s own initiative but rather the higher demand for them which might be responsible for redheaded women’s higher sexual activity and a higher number of sexual partners. It could be argued that red hair color is often perceived as less attractive (Lawson, 1971; Clayson and Maughan, 1976; Feinman and Gill, 1978; Clayson and Maughan, 1986; Clayson and Klassen, 1989; Swami and Barrett, 2011; Guéguen, 2012). But Wortham et al. (2018) showed that red hair was preferred over other hair colors more frequently than expected based on the prevalence of redheads in the studied population (both sexes preferred red hair 6% of the time, while only 3% of the female population were redheads). Given such imbalance between supply and demand, it seems that individual redheaded women should be preferred by potential mates twice more than would be proportionate to the prevalence of natural redheadedness in the female population. On the other hand, we must keep in mind that women can offset the demand by dyeing their hair red. Our unpublished data collected on 5,348 Czech women found 2.15% natural redheads and 8.73% of women who dye their hair red. Nevertheless, not all dyeing techniques can (or try to) simulate natural redheadedness, which is why it remains unclear to what extent artificial redheads affect the current or past disbalance between supply and demand for naturally redheaded women. It might also be hypothesised that for a substantive fraction of men, perceived attractiveness is not as important as the anticipated sexual permissiveness.

To test this model in the future, it would be useful to explore the sexual behavior of women who have red body hair but are not redheaded. This would differentiate between active sexual behavior of women with red hair on the one hand and behavior that is a passive consequence of mating efforts of potential suitors and prejudices about redheaded women on the other hand. We could not conduct this investigation because our sample unfortunately contained only six non-redheaded women with red body hair. Another useful approach would be to test whether naturally non-redheaded women with their hair and eyebrows dyed red are approached by men more often than when they wear their natural hair and eyebrow color. To test the proposed explanation according to which the prevailing stereotypes about more permissive redheaded women could be responsible for the observed association, future studies ought to explore men’s motives for approaching redheaded women as well as the motives of women with other hair colors for having sex.

Our analyses do not support the third model, which aimed to explain higher sexual activity and sexual desire in redheaded women by their changed self-perception. Because higher sexual desire did not mediate sexual activity in redheaded women, it is unlikely that their increased sexual activity is due to an altered self-concept that incorporates an internal conviction about the validity of stereotypes about permissive redheads. In the present study, however, we collected no data on redheaded women’s self-perception. As mentioned above, it is also possible that redheaded women have other motives for their more liberated sexual behavior apart from sexual desire – and these motives could be part of their self-concept.

We could neither prove nor disprove the fifth model, which suggests that the higher sexual activity of redheaded women is the result of a mechanism of social feedback. Based on our results, redheaded women might, in response to earlier experience with mating efforts, mostly made by males, consider higher sexual activity a social norm and react more positively in interaction with potential mates or even initiate sexual activity themselves. But to decide whether this mechanism applies, one would have to explore women’s individual motivations and opinions related to sexual behavior exhibited by women with and without red hair.

Although large cross-sectional internet studies showed worse health of redheaded individuals (Frost et al., 2017; Flegr and Sýkorová, 2019), our data did not find this pattern. We have no explanation for the absence of this effect except that our laboratory experiments may have attracted a different subpopulation, namely people of lower age and in better physical and mental health (Flegr et al., 2020b). A study made with the same sample of participants as the present study found that compared to non-redheaded individuals, redheaded subjects have higher concentrations of calcidiol, the precursor of vitamin D, and their concentration of calcidiol seems independent of the intensity of sun exposure or protection from solar radiation (Flegr et al., 2020b). That study suggested that people with red hair need less sun exposure to achieve satisfactory levels of vitamin D than non-redheaded people do. As a result, redheaded people might enjoy better health during some parts of the year, namely during autumn, winter, and spring, when the amount of UV-radiation in higher latitudes is low. At the same time, most health problems in redheaded individuals (oncological and gynaecological diseases, heart and vascular system problems, metabolic problems or fertility problems, and the like) are more common in older, rather than young people. The mean age of participants of the present study (27.3 in women and 31.8 in men) was lower than that of respondents of previous studies exploring health in redheaded people (for instance, 32.9 and 34.6 in females and 35.1 and 36.8 in males in Frost et al. (2017) and Flegr and Sýkorová (2019), respectively). The lower mean age of the present sample therefore might be why we did not find that redheaded subjects suffer from worse health.

The main limitation of this study is that the participants did not form a random, representative sample of the Czech population. We suppose that subjects who took part in the laboratory investigation may have been exposed to a sieve effect, forming a group of rather altruistic, active people in good physical and mental condition. On the other hand, whenever people have the option of refusing to participate in a study, that is, in all studies performed in accordance with the widely accepted ethical standards, the issue of non-representativeness of a sample is always present. In any case, the observed results should be verified in future by using some more representative data, for instance collected by an internet questionnaire, or by repeating the study on other samples that would not be self-selected for better health or altruism.

Another problem of the present study is the relatively low number of male participants, which may have led to false negative results for some analyses on this subset. Most associations observed in men were non-significant and the size of all effects was lower than in women. On the other hand, the size of many formally non-significant effects was relatively high, and one path analysis model for men revealed a significant positive effect of redheadedness on sexual desire. The absence of evidence of the effects of redheadedness in men therefore should not be interpreted as a non-existence of those effects.

It is fair to mention some limitations of the statistical methods used in this study. As mentioned above, the absence of statistical evidence for the existence of an effect is not a proof of its non-existence. Specifically, the path analysis did not find any evidence of a mediating role of increased sexual desire in the effect of redheadedness on sexual activity. In fact, the strength of the indirect (mediated) effect of redheadedness on sexual activity was much lower than the strength of the direct one. However, the observed differences in the strengths of direct and indirect effects could be caused not only by a real difference in effect strengths but also by differences in the precision of measurement of variables in the model. If, for example, women were willing to truthfully report the frequency of their sexual activity but reluctant to truthfully report the intensity of their sexual desire, then any statistical method, including path analysis, would necessarily underestimate the power of any mediating effect of sexual desire.

Finally, it should be noted that we tested the validity of just five models from a much larger set of all theoretically possible models. Our study seems to contradict three models and to support the other two, which naturally does not prove their validity. In fact, it is well possible that some model we did not test could be responsible for the observed associations. Specifically, sexual desire is not the only proximate reason for active initiation of sexual activities. There are many other own motives for having sex beyond sexual desire, including stress reduction, experience seeking, self-esteem boosting, striving to increase social status, seeking revenge, pursuit of resources, or pursuit of practical benefits (Meston and Buss, 2007; Meston et al., 2020). In this study, we did not ask about the reasons why redheaded women engage in sexual activities. Therefore, we cannot exclude the possibility that redheaded women actively participate in initiation of sexual activities for reasons which are not related to sexual desire.

## Conclusion

The results of this study suggest that redheaded women exhibit higher sexual desire, higher sexual activity, higher number of sexual partners, an earlier initiation of sexual life, and a higher level of sexual submissiveness. However, sexual desire does not seem to mediate the more liberated sexual behavior in redheaded women in our data. We therefore propose that an explanation of the observed association between redheadedness and certain characteristics of sexual behavior in women might be found in the stereotypical social labelling of redheaded women as being more sexually permissive, which might encourage potential mates to try and have sex with them. In the process of forming this association, redheaded women can either affirmatively respond to the courtship of potential mates or, alternatively, consider frequent sexual activities a social norm and actively initiate sexual interactions themselves. Given the lack of data regarding women’s motives for having sex and men’s motives for approaching redheaded women, we cannot consider this explanation definitive. It should be borne in mind that sexual desire is not the only one possible motive for active initiation of sexual activities. Other legitimate candidate explanations include the concentration of prenatal estrogen, faster life strategy (possibly induced by unfavorable conditions in childhood) or redheads’ own internal belief in stereotypes about themselves. Aside from that, there are other possible models which were not considered in the present study. Given the probably non-representative sample on which this study is based, our observations should be generalized with caution and our conclusions viewed rather as working hypotheses whose predictions should be tested in future, specifically designed studies.

## Data availability statement

The datasets presented in this study can be found in online repository. The names of the repository/repositories and accession number(s) can be found at: figshare: https://doi.org/10.6084/m9.figshare.21200968.v1.

## Ethics statement

The studies involving human participants were reviewed and approved by Institutional Review Board of the Faculty of Science, Charles University. The patients/participants provided their written informed consent to participate in this study.

## Author contributions

KS designed research, collected and analyzed the data, and wrote the manuscript. VF collected and analyzed the data and revised the manuscript. JH and ŠK collected the data and revised the manuscript. JF designed research and participated in the interpretation of results and writing of the manuscript. All authors contributed to the article and approved the submitted version.

## Funding

This study was supported by the Grant Agency of Charles University (project numbers 1494218 and 1310120) and Charles University Research Centre program (No. 204056).

## Conflict of interest

The authors declare that the research was conducted in the absence of any commercial or financial relationships that could be construed as a potential conflict of interest.

## Publisher’s note

All claims expressed in this article are solely those of the authors and do not necessarily represent those of their affiliated organizations, or those of the publisher, the editors and the reviewers. Any product that may be evaluated in this article, or claim that may be made by its manufacturer, is not guaranteed or endorsed by the publisher.

## Footnotes

^1^ https://osf.io/p23dk
