# Supplementary material for: Redheaded women are more sexually active than other women, but it is probably due to their suitors
Source: Front Psychol. 2022 Nov 29;13:1000753. doi: 10.3389/fpsyg.2022.1000753 (PMC9746287; doi:10.3389/fpsyg.2022.1000753)
Supplement: Supplementary file 1 [file Data_Sheet_1.docx]

## Redheaded women are more sexually active than other women, but it is probably due to their suitors

## Supplementary material

|  | | Sexual desire | | Sexual activity | | No. of sexual partners | | Age at first sexual intercourse | | Non-heterosexuality | | BDSM index | | Index of sexual dominance | |
| --- | --- | --- | --- | --- | --- | --- | --- | --- | --- | --- | --- | --- | --- | --- | --- |
|  |  | ♀ | ♂ | ♀ | ♂ | ♀ | ♂ | ♀ | ♂ | ♀ | ♂ | ♀ | ♂ | ♀ | ♂ |
| Non-redheadedness  R parameter | τ | -0.181 | -0.107 | -0.226 | -0.043 | -0.282 | -0.046 | 0.117 | 0.034 | 0.080 | -0.120 | -0.011 | 0.046 | 0.111 | 0.049 |
|  | p | **0.007*** | 0.138 | **0.001*** | 0.553 | **0.000*** | 0.561 | 0.096 | 0.649 | 0.235 | 0.127 | 0.872 | 0.577 | 0.112 | 0.557 |
| Redheadedness  *a* parameter | τ | 0.160 | 0.028 | 0.223 | 0.094 | 0.336 | 0.131 | -0.146 | -0.036 | -0.087 | 0.040 | 0.021 | -0.031 | -0.085 | 0.025 |
|  | p | **0.017*** | 0.700 | **0.001*** | 0.194 | **0.000*** | 0.098 | **0.039*** | 0.630 | 0.195 | 0.609 | 0.757 | 0.704 | 0.222 | 0.765 |
| Redheadedness observer report | τ | 0.201 | 0.062 | 0.263 | 0.038 | 0.315 | 0.158 | -0.181 | 0.019 | -0.126 | -0.007 | 0.022 | 0.048 | -0.149 | 0.042 |
|  | p | **0.003*** | 0.394 | **0.000*** | 0.604 | **0.000*** | **0.046** | **0.010*** | 0.804 | 0.062***** | 0.926 | 0.747 | 0.562 | **0.032*** | 0.617 |
| Redheadedness  self-report | τ | 0.233 | 0.066 | 0.306 | 0.088 | 0.286 | 0.102 | -0.145 | 0.082 | -0.053 | 0.063 | 0.102 | 0.110 | -0.158 | 0.035 |
|  | p | **0.000*** | 0.362 | **0.000*** | 0.226 | **0.000*** | 0.197 | **0.039*** | 0.273 | 0.434 | 0.424 | 0.141 | 0.181 | **0.023*** | 0.676 |
| Redness  of body hair | τ | 0.254 | 0.031 | 0.285 | 0.002 | 0.334 | 0.182 | -0.159 | -0.038 | -0.123 | 0.089 | 0.043 | 0.070 | -0.154 | 0.001 |
|  | p | **0.000*** | 0.667 | **0.000*** | 0.981 | **0.000*** | **0.022** | **0.024*** | 0.609 | 0.069 | 0.259 | 0.539 | 0.396 | **0.027*** | 0.993 |
| Redheadedness  in childhood | τ | 0.188 | 0.057 | 0.308 | 0.097 | 0.249 | 0.054 | -0.141 | 0.039 | -0.073 | 0.042 | 0.020 | 0.060 | -0.119 | -0.002 |
|  | p | **0.005*** | 0.434 | **0.000*** | 0.181 | **0.000*** | 0.496 | **0.048*** | 0.604 | 0.285 | 0.595 | 0.775 | 0.468 | 0.092 | 0.977 |

**Supplementary table S1**. Associations of different indicators of redheadedness with variables related to sexual life. This table shows the results of partial Kendall correlation test, with age as a covariate, analysing associations between indicators of redheadedness (first column) and variables related to sexual life in women (♀) and men (♂). Non-redheadedness R parameter and redheadedness *a* parameter are two different indicators of redheadedness measured with a spectrophotometer (for details, see Flegr *et al.*, 2020). Redness of body hair and non-heterosexuality were treated as binary variables with 0 indicating no redness and heterosexuality and 1 indicating redness of body hair and non-heterosexuality. P values under 0.05 are in bold and those under 0.001 are coded 0.000. P values significant after the correction by Benjamini-Hochberg procedure are marked with asterisks.

|  | Range | Women | | Men | |
| --- | --- | --- | --- | --- | --- |
|  |  | mean | s.d. | mean | s.d. |
| Non-redheadedness R parameter | 27.3–57.0 | 45.1 | 5.2 | 47.9 | 4.9 |
| Redheadedness *a* parameter | 1.6–15.3 | 7.4 | 3.0 | 6.2 | 2.8 |
| Redheadedness observer report | 1–6 | 2.8 | 2.1 | 2.2 | 1.9 |
| Redheadedness self-report | 1–6 | 2.5 | 1.7 | 2.2 | 1.6 |
| Redheadedness in childhood | 1–6 | 2.9 | 2.2 | 2.4 | 1.9 |
| Sexual desire: |  |  |  |  |  |
| fantasies about having sex with someone outside the partnership | 1–9 | 2.6 | 1.7 | 3.6 | 2.1 |
| sexual arousal in contact with someone outside the partnership | 1–9 | 3.8 | 2.2 | 5.4 | 2.4 |
| fantasies about having sex with someone just met | 1–9 | 2.2 | 1.6 | 3.8 | 2.3 |
| Sexual activity | 1–8 | 3.9 | 1.8 | 3.6 | 2.1 |
| No. of sexual partners of preferred sex | 1–9 | 2.6 | 1.6 | 2.5 | 1.6 |
| Age at first sexual intercourse | 0; 12–26 | 17.3 | 2.2 | 18.7 | 2.6 |
| Non-heterosexuality: |  |  |  |  |  |
| sexual attraction to people of the opposite sex | 0–100 | 94.0 | 17.5 | 89.9 | 25.7 |
| sexual attraction to people of the same sex | 0–100 | 25.3 | 29.8 | 16.0 | 31.7 |
| BDSM index; index of sexual dominance: |  |  |  |  |  |
| intensity of sexual arousal by own pain | 0–100 | 21.6 | 30.3 | 13.4 | 24.5 |
| intensity of sexual arousal by own danger | 0–100 | 15.5 | 23.9 | 10.3 | 20.5 |
| intensity of sexual arousal by own powerlessness | 0–100 | 30.6 | 32.3 | 18.0 | 26.5 |
| intensity of sexual arousal by own humiliation | 0–100 | 8.8 | 19.7 | 9.2 | 20.9 |
| intensity of sexual arousal by other’s pain | 0–100 | 11.4 | 22.6 | 19.1 | 26.6 |
| intensity of sexual arousal by other’s danger | 0–100 | 10.4 | 21.8 | 13.5 | 26.2 |
| intensity of sexual arousal by other’s powerlessness | 0–100 | 18.7 | 28.3 | 27.7 | 31.5 |
| intensity of sexual arousal by other’s humiliation | 0–100 | 9.1 | 22.5 | 17.6 | 27.5 |
| intensity of sexual arousal by violence | 0–100 | 26.1 | 31.1 | 25.2 | 30.2 |
| Age | 18–57 | 27.3 | 7.5 | 31.8 | 8.8 |
| Size of place of residence | 1–6 | 4.7 | 1.9 | 5.0 | 1.6 |
| Physical disease: |  |  |  |  |  |
| physical health problems | 0–100 | 26.6 | 17.2 | 23.8 | 16.7 |
| no. of antibiotics | 1–9 | 1.7 | 1.2 | 1.5 | 0.9 |
| no. of prescribed drugs | 1–9 | 1.5 | 0.8 | 1.4 | 0.8 |
| no. of non-prescription drugs | 1–9 | 2.0 | 1.3 | 1.7 | 1.1 |
| no. of visits to a general practitioner | 1–9 | 2.4 | 1.7 | 2.5 | 1.7 |
| no. of medical specialists | 1–9 | 3.0 | 1.8 | 2.6 | 1.3 |
| Mental disease: |  |  |  |  |  |
| mental health problems | 0–100 | 34.2 | 23.7 | 25.9 | 20.7 |
| intensity of suffering from anxieties | 0–100 | 35.1 | 30.8 | 19.3 | 21.5 |
| intensity of suffering from depressions | 0–100 | 27.4 | 31.2 | 16.0 | 20.9 |
| intensity of suffering from manias | 0–100 | 7.2 | 13.7 | 10.4 | 17.8 |
| intensity of suffering from obsessions | 0–100 | 15.2 | 22.9 | 16.8 | 20.6 |
| intensity of suffering from phobias | 0–100 | 15.5 | 20.8 | 9.6 | 17.3 |
| intensity of suffering from visual hallucinations | 0–100 | 3.1 | 8.0 | 1.4 | 2.8 |
| intensity of suffering from auditory hallucinations | 0–100 | 3.0 | 5.4 | 2.2 | 4.6 |
| intensity of suffering from burnout | 0–100 | 30.5 | 29.2 | 27.3 | 25.6 |
| intensity of suffering from headaches | 0–100 | 39.2 | 22.2 | 27.6 | 25.2 |
|  | question code | N | % | N | % |
| Redness of body hair | 0 | 69 | 63.3 | 50 | 54.9 |
|  | 1 | 40 | 36.7 | 41 | 45.1 |
| Non-heterosexuality | 0 | 95 | 0.93 | 67 | 0.88 |
|  | 1 | 7 | 0.07 | 9 | 0.12 |
| Current sexual partnership | 0 | 32 | 0.3 | 33 | 0.37 |
|  | 1 | 74 | 0.7 | 56 | 0.63 |

**Supplementary table S2**. Descriptive statistics of all variables used in the study. This table shows the means and standard deviations (s.d.) of all semi-continuous and ordinal variables and numbers (N) and percentages (%) of all binary variables for women and men. Non-redheadedness R parameter and redheadedness a parameter are two different indicators of redheadedness measured with a spectrophotometer (Flegr *et al.*, 2020). For binary variables, code 0 indicates no redness of body hair, heterosexuality, and no current sexual partnership, while code 1 indicates redness of body hair, non-heterosexuality, and being in a sexual partnership.

|  | Sex | Age | Size of place of residence | Current sexual partnership | Physical disease | Mental disease |
| --- | --- | --- | --- | --- | --- | --- |
| Non-redheadedness R parameter | **0.242*** | **0.128** | -0.044 | -0.044 | 0.036 | -0.064 |
| Redheadedness *a* parameter | **-0.170*** | -0.094 | 0.058 | 0.074 | -0.031 | 0.029 |
| Redheadedness observer report | **-0.151** | **-0.116** | 0.044 | 0.085 | 0.015 | 0.014 |
| Redheadedness self-report | -0.088 | -0.065 | 0.081 | 0.045 | -0.030 | 0.052 |
| Redness of body hair | 0.080 | 0.058 | 0.091 | 0.047 | -0.035 | 0.025 |
| Redheadedness in childhood | -0.117 | -0.066 | 0.092 | 0.088 | -0.005 | 0.013 |
| Sexual desire | **0.307*** | **0.098** | 0.089 | -0.072 | -0.029 | **0.120** |
| Sexual activity | -0.088 | 0.025 | 0.067 | **0.479*** | -0.089 | -0.019 |
| No. of sexual partners | -0.031 | -0.096 | **0.128** | **0.274*** | -0.059 | -0.076 |
| Age at first sexual intercourse | **0.222*** | **0.210*** | 0.047 | -0.008 | 0.003 | 0.039 |
| Non-heterosexuality | 0.086 | -0.053 | -0.002 | **-0.184*** | 0.065 | 0.062 |
| BDSM index | -0.009 | 0.055 | 0.024 | **0.115** | 0.021 | 0.076 |
| Sexual dominance index | **0.340*** | **0.135** | 0.018 | 0.022 | -0.029 | -0.055 |

**Supplementary table S3**. Associations of sex and potential covariates with indicators of redheadedness and variables related to sexual life. Columns 2 and 3 display Kendall’s τ. Columns to the right show partial Kendall’s τ (controlled for age). Non-redheadedness R parameter and redheadedness *a* parameter are two different indicators of redheadedness measured with a spectrophotometer (Flegr *et al.*, 2020). Sex, redness of body hair, non-heterosexuality, and current sexual partnership were treated as binary variables with 1 indicating male sex, redness of body hair, non-heterosexuality, and a current sexual partnership. Age, size of place of residence, physical disease, and mental disease were ordinal or semi-continuous variables where a higher value means a higher level of the variable in question. Associations with p values under 0.05 are in bold and those with p values under 0.005 are marked with asterisks.

| People say that on average: | Non-redheaded | Redheaded | Non-blond | Blond |
| --- | --- | --- | --- | --- |
| Light-haired women are less intelligent | 3.64 | 3.76 | 3.65 | 3.51 |
| Redheaded women are more social | 3.31 | 3.58 | 3.30 | 3.32 |
| Redheaded women are more dominant | 3.23 | 3.16 | 3.23 | 3.25 |
| Redheaded women are more often homosexual | 3.02 | 2.70 | 3.01 | 3.01 |
| Redheaded women have more sexual partners | 3.58 | 3.61 | 3.57 | 3.60 |
| Redheaded women prefer more often BDSM | 3.28 | 3.29 | 3.27 | 3.28 |

**Supplementary table S4**. Beliefs of redheaded and blond men and corresponding controls in various prejudices concerning redheaded and blond women.
The data were collected via an internet questionnaire from over 3,000 Czech and Slovak men. The critical question concerning presumed higher sexual activity of redheaded women was ‘People say that on average, they have more sexual partners:’ and the five-point scale of that question was anchored with ‘non-redheaded women’ (code 1) and ‘redheaded women’ (code 5). The table shows the arithmetic means of responses of men with traits specified in the column’s headings regarding questions specified in the rows. A mean higher than value 3 indicates that men with the trait specified in the column’s heading do actually think that people say what is described in the row. One-sample sign tests showed that responses to all questions concerning prejudices were significantly (p < 0.05) higher or lower than value 3, which would indicate no prejudice.


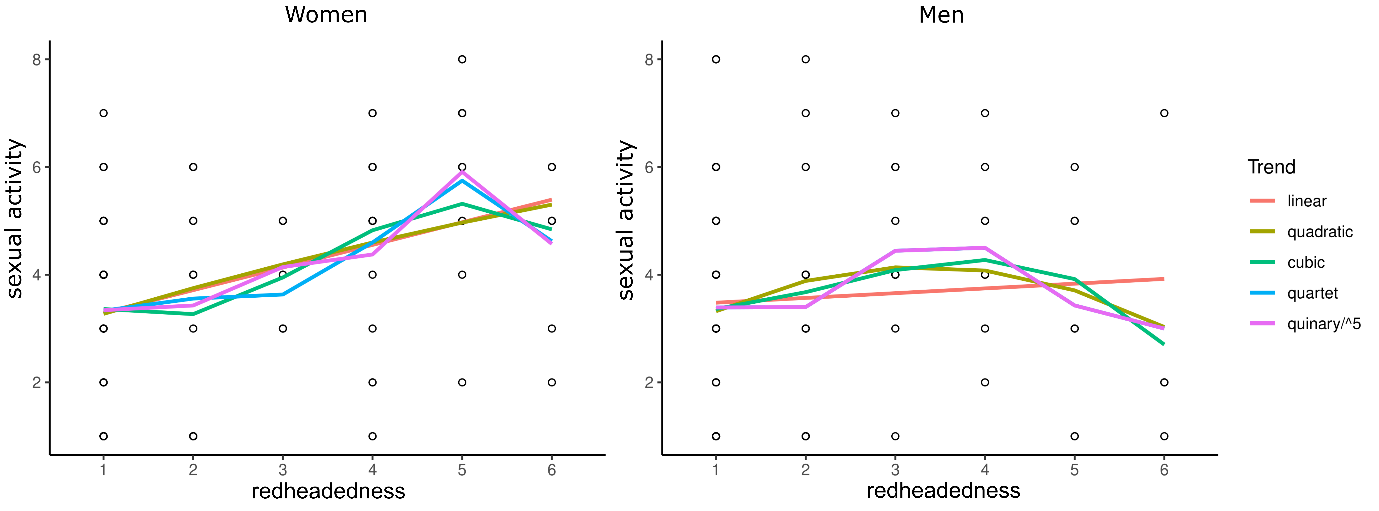


**Supplementary figure S1**. Effects of redheadedness on sexual activity in women and men. The figure visualises linear (^1) to quinary (^5) trend of association between redheadedness and sexual activity. It suggests a different association in men and women, which amounts to indirect support for a significant interaction between sex and redheadedness on sexual activity, as described in the main text.
